# Human Infrapatellar Fat Pad Mesenchymal Stem Cell-Derived Extracellular Vesicles Inhibit Fibroblast Proliferation by Regulating *MT2A* to Reduce Knee Arthrofibrosis

**DOI:** 10.1155/2023/9067621

**Published:** 2023-04-12

**Authors:** Dazhou Jia, Hui Chen, Jihang Dai, Shiping He, Yangyang Liu, Zhendong Liu, Yaxin Zhang, Xiaolei Li, Yu Sun, Qiang Wang

**Affiliations:** ^1^Department of Orthopedics, Affiliated Hospital of Yangzhou University, Yangzhou, 225001 Jiangsu, China; ^2^Department of Orthopedics, Northern Jiangsu People's Hospital Affiliated to Yangzhou University, Yangzhou, Jiangsu, China

## Abstract

Knee arthrofibrosis is one of the most serious complications of knee surgery; however, its pathogenesis is unclear, and current treatment methods have not achieved satisfactory results. Mesenchymal stem cells (MSCs) have good anti-inflammatory and antifibrotic properties, and studies have reported that human infrapatellar fat pad-derived MSCs (IPFSCs) have the advantages of strong proliferative and differentiating ability, ease of acquisition, and minimal harm to the donor. Increasing evidence has shown that MSCs function through their paracrine extracellular vesicles (EVs). Our study is aimed at exploring the effects of human IPFSC-derived EVs (IPFSC-EVs) on knee arthrofibrosis and the underlying mechanisms in vivo and in vitro. In the in vivo study, injecting IPFSC-EVs into the knee joint cavity effectively reduced surgery-induced knee arthrofibrosis in rats. In the in vitro study, IPFSC-EVs were found to inhibit the proliferation of fibroblasts in the inflammatory environment. Additionally, we screened a potential IPFSC-EV molecular target, metallothionein 2A (*MT2A*), using RNA sequencing. We found that silencing *MT2A* partially reversed the inhibitory effect of IPFSC-EVs on fibroblast proliferation in the inflammatory environment. In conclusion, IPFSC-EVs inhibit the progression of knee arthrofibrosis by regulating *MT2A*, which inhibits fibroblast proliferation in the inflammatory environment.

## 1. Introduction

Knee arthrofibrosis following knee surgery is a major challenge for most orthopedic surgeons, manifesting primarily as recurrent joint pain, knee contracture, and limitations in flexion and extension functions, all of which substantially deteriorate patients' postoperative quality of life [1]. Despite rapid advances in surgical techniques, the incidence of postoperative knee arthrofibrosis remains high. Knee arthrofibrosis occurs in 3%–10% of patients after total knee arthroplasty and in 4%–35% after cruciate ligament reconstruction [2]. Therefore, there is an urgent need for further research to solve this problem.

At present, the mechanism of arthrofibrosis following knee surgery is unclear. It is believed that the main causes of knee arthrofibrosis are excessive fibroblast proliferation and extracellular matrix production in response to various inflammatory cytokines and growth factors [3, 4]. Therefore, inhibiting excessive fibroblast proliferation in the inflammatory environment is a feasible therapeutic approach for preventing postoperative knee arthrofibrosis. It has been recently reported that inhibiting fibroblast proliferation can reduce the severity of knee arthrofibrosis [5, 6].

Many strategies for preventing arthrofibrosis in animals have been developed in recent years, including topical applications of hydroxycamptothecin, mitomycin C, and artesunate [7–9]. However, when used to prevent fibrosis, the reagents have been shown to damage surrounding normal tissues, interfere with local tissue healing, and cause toxic adverse effects after reagent absorption [10]. Therefore, there is an urgent need to develop safer and more effective treatments for knee arthrofibrosis.

Studies have reported that mesenchymal stem cells (MSCs) have positive therapeutic effects on fibrotic diseases, such as pulmonary, hepatic, and renal fibrosis [11–13]. However, the use of MSC transplantation is greatly limited due to issues with rejection reactions and low survival rates [14]. MSCs function through their paracrine extracellular vesicles (EVs) [15]. EVs contain abundant mRNA, miRNA, lipids, and active proteins, which play a crucial role in signal transduction between cells [16–18]. Compared with MSCs, MSC-derived EVs (MSC-EVs) are noncellular components that can enrich the active components of source cells, have lower immunogenicity, can overcome the hidden danger of tumorigenicity after MSC transplantation, and have promising application prospects [19]. MSC-EVs has been shown to promote cartilage repair and alleviate osteoarthritis (OA) [20], and a previous study demonstrated that rat adipose MSC-EVs could facilitate cartilage injury repair and have better anti-OA effects by increasing chondrocyte viability and migration and suppressing cell apoptosis [21]. MSC-EVs have also been shown to prevent the development of OA via the circHIPK3/miR-124-3p/MYH9 axis [22]. Among MSCs, human infrapatellar fat pad-derived MSCs (IPFSCs) are an ideal donor choice owing to their strong proliferative and differentiative ability, abundant sources, ease of acquisition (partial resection of the infrapatellar fat pad is required in knee arthroplasty), and minimal harm to the donor [23]. However, it remains unknown whether IPFSC-EVs can inhibit the progression of knee arthrofibrosis. We hypothesized that the use of IPFSC-EVs could be a novel approach for preventing postoperative knee arthrofibrosis.

In this study, we established a rat model of surgery-induced knee arthrofibrosis, injected IPFSC-EVs into the knee joint cavity, and collected specimens for histological preparation 4 weeks after surgery. Using hematoxylin and eosin staining, Masson staining, and immunohistochemistry, we found that IPFSC-EVs reduced the expression of proinflammatory factors (IL-6 and TNF-*α*) and inhibited the degree of postoperative knee arthrofibrosis. Using transcriptomic sequencing, we screened metallothionein 2A (*MT2A*), a potential target gene of IPFSC-EVs, for its ability to inhibit knee arthrofibrosis. Studies have shown that *MT2A* plays an important role in regulating cell proliferation and inflammatory responses [24–27]. Therefore, exploring the effects of IPFSC-EVs on fibroblast proliferation in the inflammatory environment and the role of *MT2A* in this process is expected to provide new intervention targets for treating fibrotic diseases in clinical practice.

## 2. Materials and Methods

### 2.1. Animals

The study was approved by the Animal Ethics Committee of Yangzhou University. A total of 48 male Sprague–Dawley rats weighing 250–300 g were purchased from the Experimental Animal Center of Yangzhou University (Yangzhou, China). Before the experiment, all rats were carefully housed in the animal room at a constant temperature (24 ± 0.5°C) and humidity (50%–60%). The rats were randomly divided into four groups (*n* = 12 per group): a phosphate-buffered saline (PBS) control group and three IPFSC-EV intervention groups (10^9^, 5 × 10^9^, and 10^10^ particles/mL). The IPFSC-EV concentrations used were selected based on a previous study [28].

### 2.2. Establishing the Animal Model and Injecting IPFSC-EVs into the Knee Joint Cavity

The knee arthrofibrosis model was established as described in a previous study [6]. Briefly, after the rats were fully anesthetized, a cortical bone area of approximately 3 × 3 mm^2^ was removed to expose the cancellous bone. After satisfactory hemostasis, the incision was sutured in layers. Finally, Kirschner wires were used to fix the knee joint in a fully flexed position. Penicillin (50 mg/kg) was administered for 3 consecutive days after surgery to prevent infection. From the first postoperative week, 10 *μ*L of IPFSC-EVs at various concentrations (10^9^, 5 × 10^9^, and 10^10^ particles/mL) was injected into the knee joint cavity under ultrasound guidance. The control group was injected the same dose of PBS twice a week for 4 weeks.

### 2.3. Histological Analysis

Rats were euthanized 4 weeks after establishing the model, following which the knee joint specimens were collected for histological analysis. The specimens were fixed in 4% paraformaldehyde for 1 week, fully decalcified in ethylenediaminetetraacetic acid, and embedded in paraffin. Subsequently, the four groups of specimens were cut into sections and stained with hematoxylin and eosin to observe the degree of fibrosis. The density and content of collagen (collagen I and III) in the fibrotic tissues were observed via Masson staining and immunohistochemistry, respectively. The content of proinflammatory cytokines (IL-6 and TNF-*α*) and fibrosis marker (*α*-SMA) in the fibrotic tissues were also observed via immunohistochemistry.

### 2.4. Cell Isolation and Culture

The human fibroblast cell line was purchased from ScienCell Research Laboratories (Carlsbad, CA, USA). Fibroblasts were cultured in Dulbecco's Modified Eagle Medium ([DMEM]; Gibco, CA, USA) supplemented with 10% fetal bovine serum ([FBS]; Clarkbio, VA, USA) and 1% penicillin–streptomycin ([PS]; Beyotime, Shanghai, China) in a humidified atmosphere with 5% CO_2_. Human IPFSCs were derived from patients undergoing knee arthroplasty. All patients participating in this study provided written informed consent, and the study was approved by the Ethics Committee of Northern Jiangsu People's Hospital affiliated with Yangzhou University. The fragments of infrapatellar fat pad were digested in PBS containing 0.2% collagenase type I (Beyotime, Shanghai, China) at 37°C for 10 h. The cell suspension was then filtered through a 40 *μ*M cell strainer and resuspended in DMEM supplemented with 10% FBS and 1% PS.

### 2.5. Detection of IPFSC Surface Antigens Using Flow Cytometry

IPFSC surface antigens were detected using the Human MSC Analysis Kit (BD Biosciences, USA) according to the manufacturer's instructions. After reaching 90% confluency, the cells were digested with trypsin, and the cell concentration was adjusted to 10^7^ cells/mL. Anti-CD90, anti-CD44, anti-CD105, and anti-CD73 antibodies were then added separately to the cell suspension. After incubation at room temperature for 30 min in the dark, the cells were detected using flow cytometry.

### 2.6. Identification of Trilineage Differentiation of IPFSCs

This experiment was performed using the human adipose MSC induction and differentiation kit (Cyagen Biosciences, Guangzhou, China) strictly according to the manufacturer's instructions. The osteogenic, adipogenic, and chondrogenic induction media were prepared in advance, and the induction was performed for up to 3 weeks. Mineral content of the cultures was determined using Alizarin Red staining, endoacidic mucopolysaccharide content in cartilage tissue was determined using Alician Blue staining, and lipid accumulation was determined using Oil Red O staining.

### 2.7. Isolation and Identification of IPFSC-EVs

After reaching 60% confluency, the IPFSCs were washed twice with PBS. The cell culture medium was then replaced with serum-free medium for EVs (Umibio, Shanghai, China). After culturing for 48 h, the conditioned IPFSC medium was collected to isolate the EVs, which were extracted using ultracentrifugation. The morphology of IPFSC-EVs was observed using transmission electron microscopy. The particle size distribution and concentration of IPFSC-EVs were determined using the NanoSight LM10 instrument (Malvern, UK). Protein markers of EVs were detected by western blot, including CD63 and CD81. Fibroblasts were incubated with PKH26- (red) labeled IPFSC-EVs, and the uptake of IPFSC-EVs by fibroblasts was observed under a confocal laser scanning microscope.

### 2.8. Cell Activity Detection

The fibroblast suspension was added to a 96-well plate at a density of 5000 cells/well. To simulate the cellular inflammatory environment, the inflammatory fibroblast model was established using interleukin (IL)-1*β*. The fibroblasts were treated with 10 ng/mL of IL-6 and IPFSC-EVs at various concentrations (10^8^, 5 × 10^8^, and 10^9^ particles/mL) for 24 h. Subsequently, 10 *μ*L of Cell Counting Kit-8 (CCK-8) buffer was added to each well, and the plate was incubated at 37°C for 2 h. The absorbance of cells was measured at 450 nm using a microplate reader.

### 2.9. RNA Sequencing for Fibroblasts

Fibroblasts in the inflammatory environment were treated with IPFSC-EVs (10^9^ particles/mL) for 24 h, and total RNA was extracted using TRIzol (Tiangen, Beijing, China). After the RNA samples had passed the quality control, mRNAs with polyA tails were enriched using Oligo (dT) magnetic beads. Next, a cDNA library was created, and after it was qualified, on-machine sequencing was performed, with the DESeq algorithm used to calculate differentially expressed genes. Using |log2(FoldChange)| > 0 and Padj < 0.05 as the screening conditions, a volcano plot of genes differentially expressed between the IPFSC-EV and control groups was constructed. Subsequently, Gene Ontology (GO) analysis was performed to help elucidate the biological implications of the differentially expressed genes, including biological processes, cellular composition, and molecular functions, and Kyoto Encyclopedia of Genes and Genomes (KEGG) pathway analysis was performed to identify significantly enriched pathways affected by the differentially expressed genes. Combined with the specific functions of these genes, the molecular target of IPFSC-EVs was identified.

### 2.10. Flow Cytometry to Detect Fibroblast Proliferation

This experiment was performed using the Cell Cycle Detection kit (keyGEN BioTECH, Nanjing, China). Fibroblasts were treated with IPFSC-EVs (10^9^ particles/mL) for 24 h in an inflammatory environment, washed with PBS, and then fixed in 70% ice-cold ethanol overnight. The fibroblasts were then rewashed with PBS, and each sample was resuspended in 0.5 mL of propidium iodide and allowed to stand for 30 min in the dark. The samples were then assessed using flow cytomtery, and the data were analyzed using FlowJo software.

### 2.11. EdU Cell Proliferation Assay

This assay was performed using the EdU Cell Proliferation Kit (keyGEN BioTECH). Fibroblasts in the inflammatory environment were treated with IPFSC-EVs (10^9^ particles/mL) for 24 h, 50 *μ*M of EdU working fluid was added to each well, following which the wells were allowed to stand for 2 h. The cells were fixed with 4% paraformaldehyde for 30 min and then permeabilized with PBS containing 0.5% Triton X-100. After counterstaining with Hoechst 33342 reaction solution, EdU-positive cells and nuclear staining were observed under a fluorescence microscope.

### 2.12. Western Blot Assay

A western blot was performed as previously described [29]. Briefly, cells were lysed on ice using radioimmunoprecipitation assay buffer (Beyotime, Shanghai, China), and the protein content was determined using the bicinchoninic acid protein assay kit (Beyotime, Shanghai, China). Separating gels with different concentrations of acrylamide (10%–15%) were prepared according to the molecular weight of the target proteins. After separation via electrophoresis, the proteins were transferred onto polyvinylidene difluoride membranes (Millipore, Bedford, MA). The membranes were then placed in in 5% skimmed milk for 2 h and incubated with primary antibodies (antiproliferating cell nuclear antigen [PCNA], anti-cyclin D1, anti-IL-6, anti-tumor necrosis factor [TNF]-*α*, and anti-*β*-actin, which were purchased from Cell Signaling Technology, and anti-*MT2A*, purchased from Abcam) overnight at 4°C, followed by incubation with the corresponding secondary antibodies for 2 h at room temperature. Finally, protein signals were detected using chemiluminescence by visualizing the membranes under the BIO-RAD ChemiDoc XRS imaging system.

### 2.13. Reverse Transcription–Polymerase Chain Reaction (RT–PCR)

Fibroblasts were treated with IL-1*β* (10 ng/mL) and IPFSC-EVs (10^9^ particles/mL) for 24 h, and total RNA was extracted using TRIzol (Tiangen, Beijing, China), after which cDNA was synthesized using the HiScript III RT SuperMix for qPCR (+gDNA wiper) kit (Vazyme, Nanjing, China). Subsequently, RT–PCR was performed using the AceQ Universal SYBR qPCR Master Mix kit (Vazyme, Nanjing, China). Gene expression levels were determined using the 2^−∆∆CT^ method. The primer sequences used for RT–PCR are listed in Table 1.

### 2.14. Lentiviral Vector Infection

Lentiviral-mediated permanent transfection was performed to silence *MT2A*. Fibroblasts were incubated with the lentiviral vector for RNA interference (RNAi) of *MT2A* (LVMT2A) and negative control RNAi (Genechem Co., Ltd., Shanghai, China) at a multiplicity of infection of 20 for 16 h according to the manufacturer's instructions. After transfection for 72 h, the transfection efficiency was determined using fluorescence microscopy. Lentiviral-transfected fibroblasts were cultured with 2 *μ*g/mL puromycin (Sigma, USA) for 48 h. RT–PCR and western blot analyses were performed to determine the gene and protein expression levels of *MT2A*, respectively. Stably transfected fibroblasts with silenced *MT2A* were used for subsequent experiments.

### 2.15. Statistical Analysis

The data for this study are presented as mean ± standard deviation (SD). The statistical analysis was performed using the SPSS 19.0 statistical software. Pairwise comparisons were performed using Student's *t*-test, whereas multiple-group comparisons were performed using one-way analysis of variance. A *P* value of <0.05 was considered statistically significant.

## 3. Results

### 3.1. Identification of IPFSCs and IPFSC-EVs

**Figure 1:**
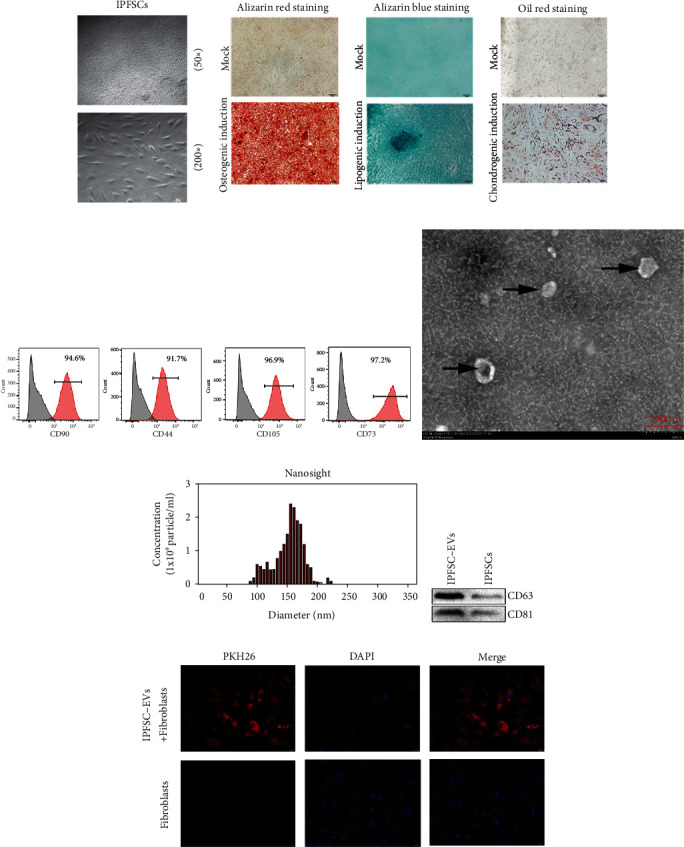
Identification of infrapatellar fat pad mesenchymal stem cells (IPFSCs) and IPFSC-EVs. (a) IPFSCs were isolated and cultured, showing a spindle-like shape under optical microscopy. (b) IPFSCs had adipogenic, osteogenic, and chondrogenic differentiation ability. (c) Characteristic antigens of IPFSCs detected using flow cytometry. (d) IPFSC-EVs showed a typical disc-shaped bilayer membrane structure under transmission electron microscopy. (e) NanoSight analysis showing the diameter range of the extracted EVs. (f) Western blot showing the expression levels of the characteristic EV proteins CD63 and CD81. (g) After PKH26 labeling of IPFSC-EVs, uptake of IPFSC-EVs by fibroblasts was observed under fluorescence microscopy.

The human IPFSCs showed a typical spindle-like shape and swirl arrangement under optical microscopy (Figure 1(a)). The trilineage differentiation of human IPFSCs was identified using Alizarin Red staining, which revealed dark red calcium nodules and several osteoblasts. Alcian Blue staining was positive, indicating the presence of endoacidic mucopolysaccharide in cartilage tissue. Oil Red O staining revealed visible fat droplets as well as several adipocytes (Figure 1(b)). Flow cytometry detection of cell surface antigens revealed high expression of positive stem cell markers (CD44, CD73, CD90, and CD105) in the isolated cells (Figure 1(c)). Considering the abovementioned results, these findings are concordant with the characteristics of MSCs. The EVs obtained using ultracentrifugation had a typical disc-shaped bilayer membrane structure and a diameter of approximately 100 nm, as revealed through transmission electron microscopy (Figure 1(d)). NanoSight analysis revealed that the EVs ranged from 50 to 200 nm in size and their concentration was approximately 10^10^ particles/mL (Figure 1(e)). Western blot analysis revealed that the extracted EVs highly expressed the EV surface markers CD63 and CD81 (Figure 1(f)). Based on the abovementioned results, we confirmed that the extracted EVs were IPFSC-EVs. In addition, Fibroblasts were incubated with PKH26- (red) labeled IPFSC-EVs, and the uptake of EVs by fibroblasts was observed under the microscope after 24 h. Red fluorescence was observed in fibroblasts (Figure 1(g)). The above results indicated that fibroblasts could uptake IPFSC-EVs.

### 3.2. IPFSC-EVs Delay the Progression of Knee Arthrofibrosis in Rats

**Figure 2:**
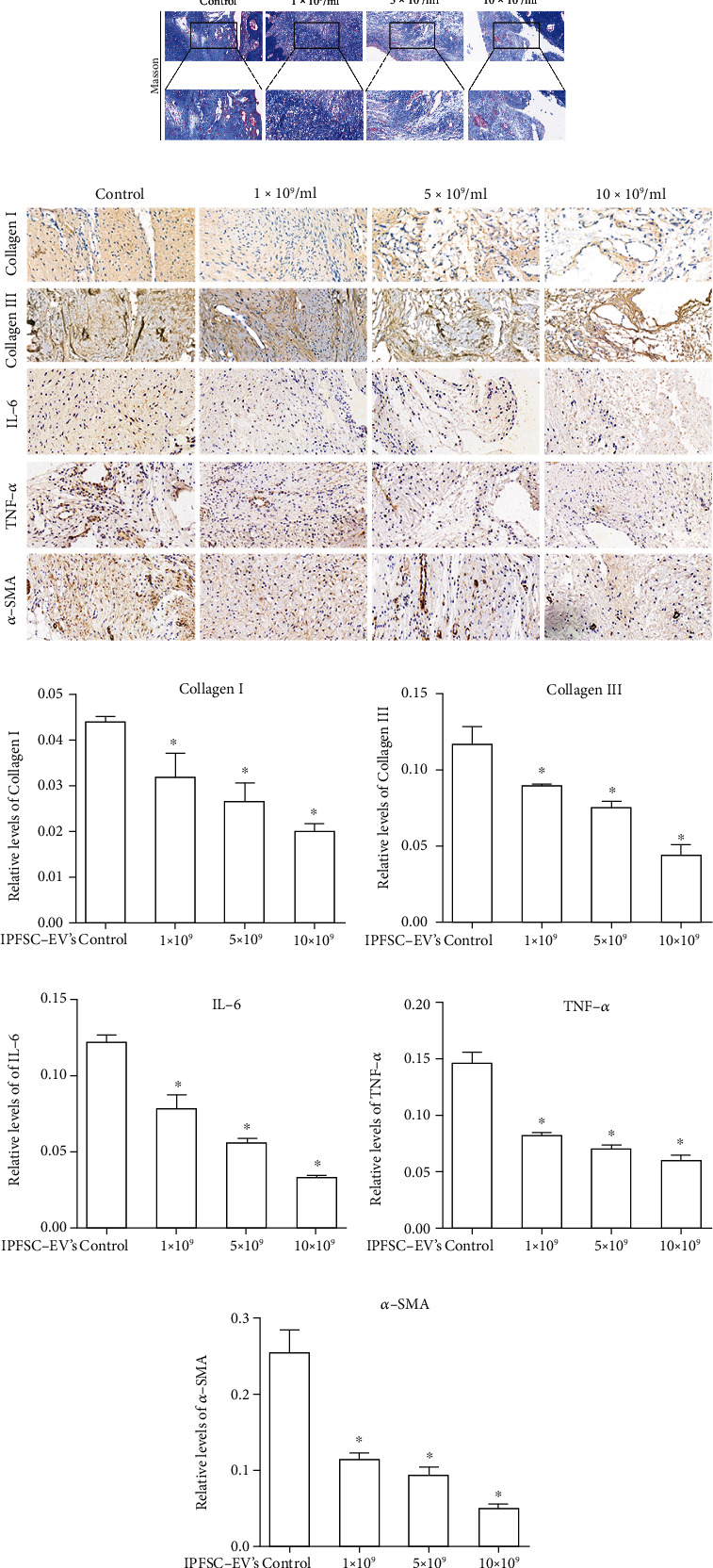
IPFSCs-EV treatment reduced the severity of knee arthrofibrosis in rats. (a) Hematoxylin and eosin staining showed that IPFSC-EVs reduced the degree of fibrosis and fibroblast number in the knee joint cavity. Masson staining showed that IPFSC-EVs reduced collagen content in the fibrotic tissues. (c and d) Immunohistochemical staining of collagens I and III revealed that IPFSC-EVs reduced collagen content in the fibrotic tissues in a concentration-dependent manner. (e and f) Immunohistochemical staining of IL-6 and TNF-*α* showed that IPFSC-EVs reduced the expression of proinflammatory factors in the fibrotic tissues in a concentration-dependent manner. (g) Immunohistochemical staining of *α*-SMA also revealed that IPFSC-EVs reduced the degree of fibrosis in the knee joint cavity in a concentration-dependent manner.

To examine the inhibitory effect of IPFSC-EVs on knee arthrofibrosis in rats, we injected different concentrations of IPFSC-EVs (10^9^, 5 × 10^9^, and 10^10^ particles/mL) into the knee joint cavity to observe whether IPFSC-EVs could delay the progression of knee arthrofibrosis. Hematoxylin and eosin staining revealed that the control group had dense fibrotic tissues, and the IPFSC-EV groups exhibited significantly less degree of fibrosis than the control group. With increasing IPFSC-EV concentration, the degree of fibrosis in the knee joint cavity was gradually reduced. Similarly, Masson staining revealed that the collagen content in the knee joint cavity gradually decreased with the increase in IPFSC-EV concentration (Figure 2(a)). Immunohistochemical analysis revealed that IPFSC-EVs decreased the expression levels of collagens I and III in the knee joint cavity in a concentration-dependent manner (Figures 2(c) and 2(d)), which was consistent with the results of Masson staining. In addition, immunohistochemical analysis also revealed that IPFSC-EVs could reduce the content of proinflammatory cytokines (IL-6 and TNF-*α*) and fibrosis marker (*α*-SMA) in a concentration-dependent manner (Figures 2(e)–2(g)). Taken together, these findings indicated that IPFSC-EVs could effectively inhibit the progression of knee arthrofibrosis in rats.

### 3.3. Construction of a Cellular Inflammatory Model and Detection of Fibroblast Proliferation Activity via CCK8 Assay

**Figure 3:**
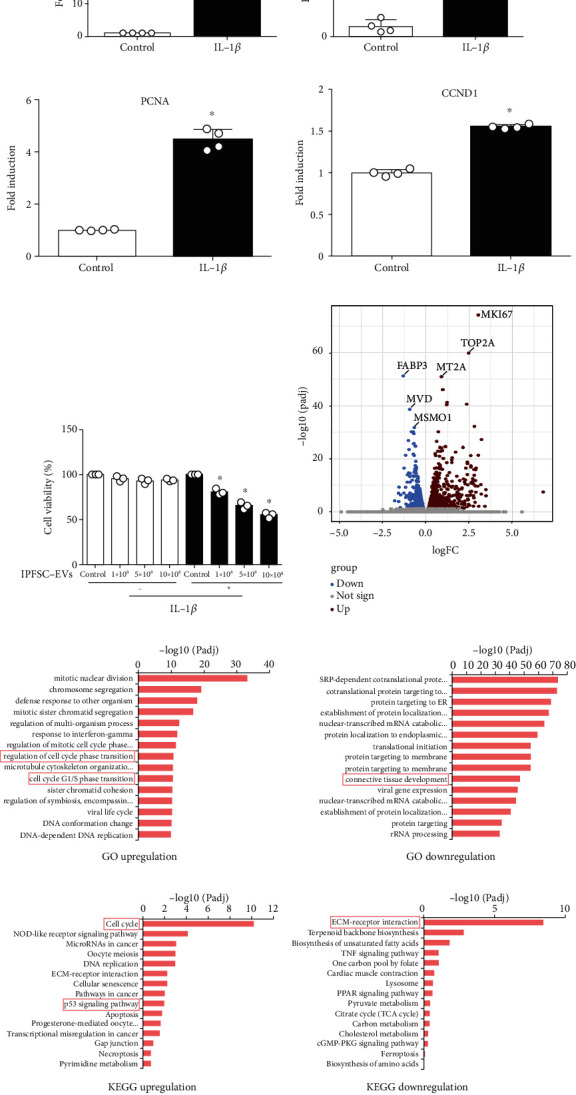
Construction of a fibroblast inflammatory model and screening of molecular targets for inhibiting knee arthrofibrosis. (a) Increased expression of the inflammatory factors IL-6 and TNF-*α* after IL-1*β* stimulation. (b) Increased expression of the proliferation-related genes *PCNA* and *CCND1* after IL-1*β* stimulation. (c) IL-1*β* (10 ng/mL) was combined with various IPFSC-EV concentrations to react with fibroblasts for 24 h. CCK-8 assay revealed that fibroblast viability was suppressed in a concentration-dependent manner. (d) Volcano plot showing genes that were differentially expressed between IPFSC-EVs and controls. (e) GO analysis showing upregulated cell cycle phase transition and cell cycle G1/S phase transition and downregulated connective tissue development. (f) KEGG pathway analysis showing upregulated cell cycle and p53 signaling pathway and significantly downregulated extracellular matrix–receptor interactions. All data are presented as mean ± SD. ^∗^*P* < 0.05 compared with the control group.

IL-1*β* is a key mediator of the inflammatory response and the development of pathological conditions that lead to chronic inflammation. Studies have reported that IL-1*β* could drive the inflammatory phenotype of fibroblasts and play a significant role in the fibrosis process [30, 31]. To simulate the inflammatory environment of fibroblasts, we used a low concentration of IL-1*β* (10 ng/mL) to create a cellular inflammatory model. The model's reliability was validated using RT–PCR, and results showed that the cellular inflammatory model could promote the expression of inflammatory cytokines (IL-6 and TNF-*α*) (Figure 3(a)). We also found that the low concentration-IL-1*β* inflammatory environment could promote the expression of fibroblast proliferation-related genes (*CCND1* and *PCNA*) (Figure 3(b)). In other words, inflammatory stimulation could promote fibroblast proliferation, which is consistent with the current theory of the mechanism of fibrosis formation [3, 4]. Additionally, we verified the effects of IPFSC-EVs on fibroblast viability in noninflammatory and inflammatory environments using CCK8 assay. The results revealed that IPFSC-EVs had no significant effect on fibroblast proliferation in the noninflammatory environment but could significantly inhibit fibroblast proliferation in the inflammatory environment in a concentration-dependent manner, with an IPFSC-EV concentration of 10^9^ particles/mL having the greatest inhibitory effect (Figure 3(c)). Therefore, we used 10^9^ particles/mL of IPFSC-EVs for subsequent experiments.

### 3.4. Screening of Molecular Targets for Inhibiting Knee Arthrofibrosis Using RNA-Seq

**Figure 4:**
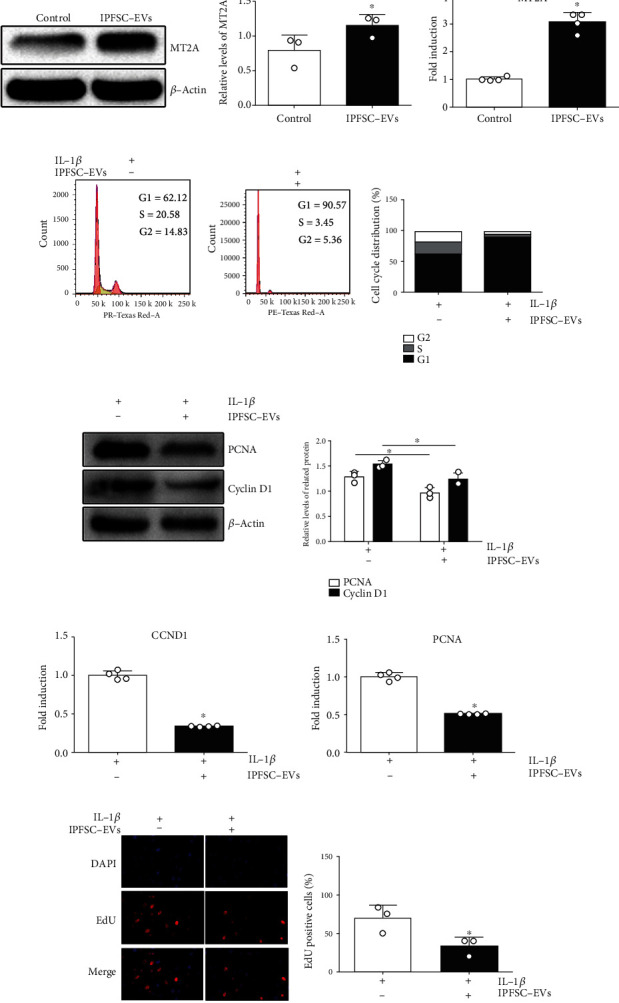
IPFSC-EVs inhibited fibroblast proliferation in the inflammatory environment. (a) Western blot and RT–PCR showing that IPFSC-EVs could promote *MT2A* expression. (b) Cell cycle analysis showing that IPFSC-EVs could arrest fibroblasts in the G1 phase in the inflammatory environment and inhibit cell cycle progression. (c and d) Western blot and RT–PCR results showing downregulated mRNA and protein expression levels of the proliferation-related genes *CCND1* and *PCNA* after IPFSC-EV treatment of fibroblasts. (e) EdU cell proliferation assay showing a significantly lower proportion of EdU-positive cells in total cells after IPFSC-EV treatment of fibroblasts in the inflammatory environment for 24 h. All data are presented as mean ± SD. ^∗^*P* < 0.05 compared with the control group.

To screen potential molecular targets of IPFSC-EVs that can be used to prevent knee arthrofibrosis, we treated fibroblasts with IPFSC-EVs (10^9^ particles/mL) in the inflammatory environment for 24 h and then performed transcriptomic sequencing (RNA-seq). The volcano plot revealed differentially expressed genes between the IPFSC-EV and control groups (Figure 3(d)). GO analysis revealed that processes such as the regulation of cell cycle phase transition and cell cycle G1/S phase transition were upregulated, whereas processes such as connective tissue development were downregulated (Figure 3(e)). KEGG pathway analysis revealed that the cell cycle and p53 signaling pathway were upregulated and that extracellular matrix–receptor interaction was significantly downregulated (Figure 3(f)). By combining the results of GO and KEGG pathway analyses with specific functions of these genes, we identified the significantly upregulated gene *MT2A* as being related to the progression of knee arthrofibrosis. We verified the reliability of the obtained molecular targets using western blot and RT–PCR. The results revealed that the mRNA and protein expression levels of *MT2A* were significantly increased after the fibroblasts were treated with IPFSC-EVs (Figure 4(a)).

### 3.5. IPFSC-EVs Inhibit Fibroblast Proliferation in the Inflammatory Environment

The effects of IPFSC-EVs on fibroblast proliferation in the inflammatory environment were verified using flow cytometry, western blot, RT–PCR, and the EdU cell proliferation assay. In the flow cytometry analysis, the proportion of fibroblasts in the G1 phase increased after treatment with IPFSC-EVs, whereas that of fibroblasts in the S and G2 phases significantly decreased, indicating that IPFSC-EVs could arrest fibroblasts in the G1 phase and inhibit their proliferation (Figure 4(b)). In the western blot and RT–PCR analyses, the mRNA and protein levels of the proliferation-related genes *CCND1* and *PCNA* were decreased (Figures 4(c) and 4(d)). The decrease in cyclin D1 expression indicated that the cell cycle was inhibited, while the decrease in PCNA expression indicated that cellular DNA synthesis was reduced, both indicating inhibition of cell proliferation. In the EdU cell proliferation assay, the proportion of EdU-positive cells in total cells was significantly reduced (Figure 4(e)), which also indicated that IPFSC-EVs could effectively inhibit fibroblast proliferation. In summary, IPFSC-EVs could inhibit fibroblast proliferation in the inflammatory environment.

### 3.6. IPFSC-EVs Inhibit Fibroblast Proliferation in the Inflammatory Environment by Regulating *MT2A*

**Figure 5:**
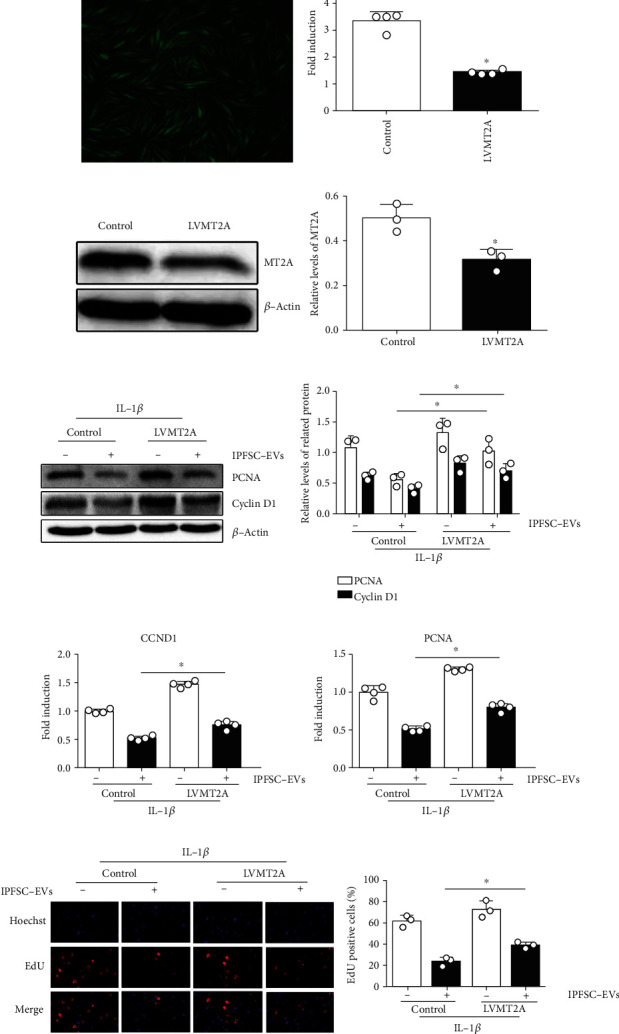
IPFSC-EVs inhibited fibroblast proliferation in the inflammatory environment by regulating *MT2A*. (a) Cell transfection efficiency under fluorescence microscopy. (b and c) Western blot and RT–PCR analyses showing decreased gene and protein expression of *MT2A* in the virus strain, indicating successful *MT2A* silencing. (d and e) Western blot and RT–PCR analysis showing the partially reversed tendency of IPFSC-EVs to inhibit *CCDN1* and *PCNA* expression after silencing *MT2A*. (f) EdU cell proliferation assay showing the partially reversed ability of IPFSC-EVs to inhibit DNA synthesis in fibroblasts after silencing *MT2A*. All data are presented as mean ± SD. ^∗^*P* < 0.05 compared with the control group.

**Figure 6:**
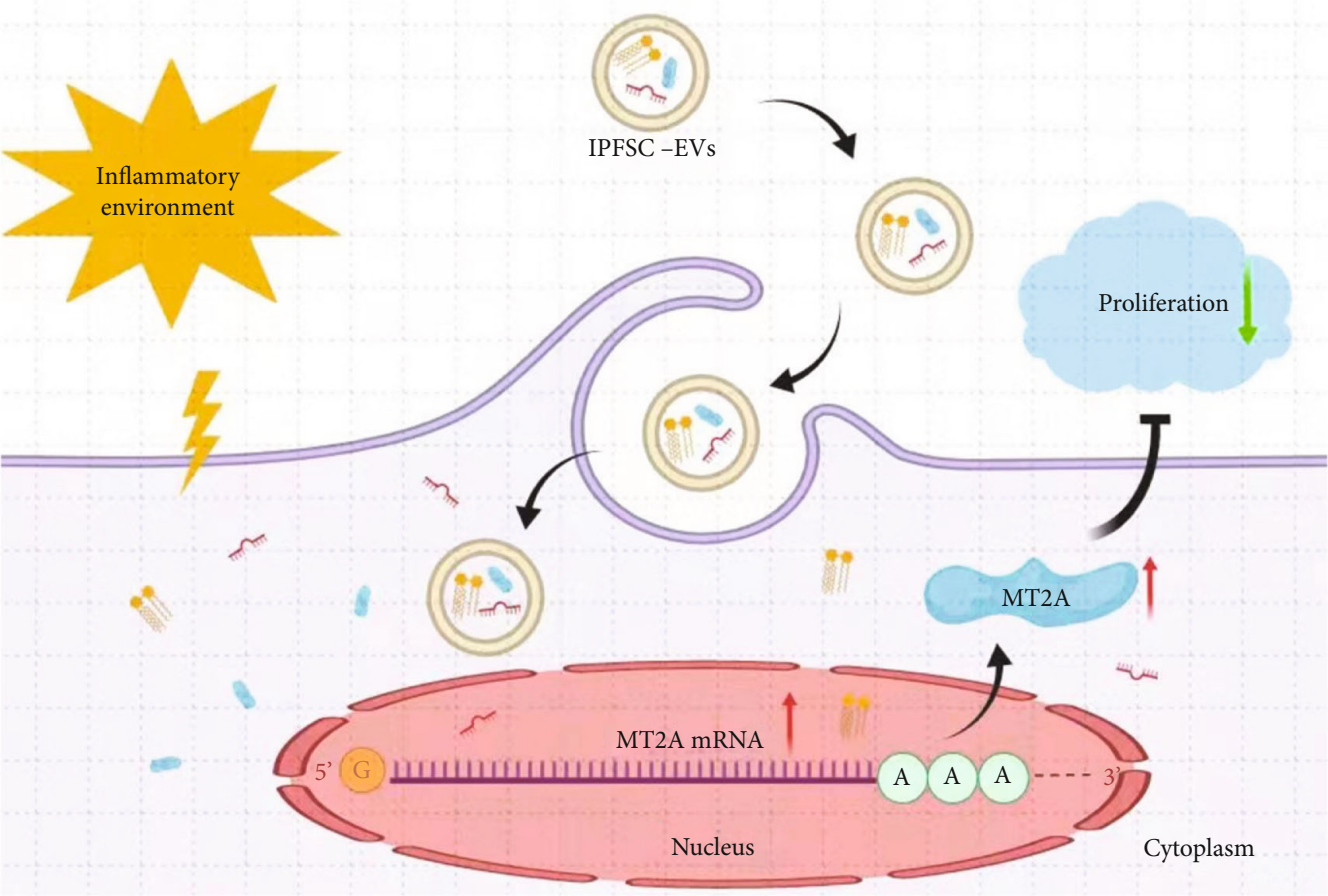
A proposed underlying mechanism of IPFSC-EVs in attenuating the progression of knee arthrofibrosis.

To further explore the underlying mechanism of how IPFSC-EVs inhibit fibroblast proliferation in the inflammatory environment, *MT2A* was silenced via lentiviral vector transfection. The transfection efficiency was approximately 80% as observed under fluorescence microscopy, and the cells grew well (Figure 5(a)). Western blot and RT–PCR analyses revealed that when compared with the negative control virus group, the LVMT2A group could significantly silence the mRNA and protein expression of *MT2A*, thus meeting the requirements of subsequent experiments (Figures 5(b) and 5(c)). After silencing *MT2A*, RT–PCR and western blot analyses revealed that the inhibitory effect of IPFSC-EVs on the mRNA and protein expression levels of proliferation-related genes (*CCND1* and *PCNA*) were partially reversed (Figures 5(d) and 5(e)). Moreover, the EdU cell proliferation assay showed that the inhibitory effect of IPFSC-EVs on the proportion of EdU-positive cells was also partially reversed (Figure 5(f)). Taken together, these results demonstrated that IPFSC-EVs inhibited fibroblast proliferation in the inflammatory environment by regulating *MT2A* (Figure 6).

## 4. Discussion

To the best of our knowledge, this is the first study to show that IPFSC-EVs can effectively reduce the severity of surgery-induced knee arthrofibrosis in rats, and that this effect might be mediated by regulating the molecular target *MT2A*, thereby inhibiting fibroblast proliferation in an inflammatory environment. Our findings suggest a possible strategy for preventing the development of knee arthrofibrosis following orthopedic surgery.

Knee arthrofibrosis is extremely distressing to patients as it causes persistent pain, limited joint mobility, and even severe disability [32]. These symptoms can lead to an inability to perform daily living activities and have a profoundly detrimental effect on physical and mental health [33]. Current prevention and treatment strategies primarily focus on improving surgical techniques and early rehabilitation exercises; however, the clinical results obtained to date have been unsatisfactory [34].

Excessive fibroblast proliferation has recently been shown to be an important factor in the progression of fibrotic diseases [35–37]. When the body is injured, the tissues undergo an inflammatory response, which in turn produces various stimuli to promote the activation and proliferation of fibroblasts, resulting in the secretion of a large amount of extracellular matrix and eventually leading to the formation of local fibrosis [38, 39]. Therefore, inhibiting fibroblast proliferation remains the primary focus of research on preventing and treating articular fibrosis.

Several studies have confirmed that MSC-derived EVs can inhibit the formation of fibrosis [40–42]. One study reported that human bone marrow MSC-derived EVs (BMSC-EVs) could reduce collagen deposition, inhibit inflammation, and increase hepatocyte regeneration, thereby effectively alleviating liver fibrosis in rats [43]. BMSC-EVs can also effectively alleviate silica-induced pulmonary fibrosis, including inhibiting the profibrotic transforming growth factor-*β*1 and downregulating the expression level of the fibrosis marker protein, alpha-smooth muscle actin [44]. Moreover, adipose MSC-derived EVs have been shown to effectively inhibit the proliferation and migration of hypertrophic scar fibroblasts and reduce the expression levels of collagen I, collagen III, and alpha-smooth muscle actin [45]. Similarly, our study also confirmed that IPFSC-EVs can effectively reduce the number of fibroblasts and expression levels of collagens I and III in fibrotic tissues. To further explore the underlying mechanism, *MT2A* was identified using RNA-seq, and it was implicated to be involved in the progression of knee arthrofibrosis.

Human metallothioneins (MTs) are a class of low-molecular-weight proteins that are widely present in mammals, higher plants, and certain prokaryotes and are a large family of cysteine-rich molecules [46]. MTs have high affinity for metal ions and reactive oxygen species and have been reported to exert protective effects in various animal models, including lipopolysaccharide-induced lung injury, rheumatoid arthritis, multiple sclerosis, coagulopathy, ethanol-induced gastroduodenal mucosal injury, and gastritis caused by *Helicobacter pylori* [47–52]. MTs have four isoforms—MT1, MT2, MT3, and MT4—and *MT2A* is an important member of the MT family. In recent years, studies have shown that *MT2A* is closely related to the regulation of cell proliferation [53, 54]. *MT2A* expression was found to be decreased in gastric cancer cell lines and primary tumor tissues, and *MT2A* upregulation could significantly decrease cyclin D1 expression in gastric cancer cells, further indicating that *MT2A* upregulation could inhibit gastric cancer cell proliferation [53]. These results are consistent with our study result that *MT2A* upregulation can inhibit cell proliferation. However, *MT2A* was highly expressed in breast cancer tissues in a previous study, and the mRNA content of *MT2A* was positively correlated with the Ki-67 index (a marker of cell proliferation activity), which supported the hypothesis that high *MT2A* expression could promote breast cancer cell proliferation [54]. *MT2A* expression differs significantly among cells derived from tissues of various diseases, and its effect on the proliferation of tissue-derived cells also differs considerably. Therefore, our findings might thus provide a foundation for future research.

In the in vivo study, we established a model of surgery-induced knee arthrofibrosis in rats. The histological analysis of knee fibrotic tissues stained with hematoxylin and eosin indicated that IPFSC-EVs could significantly reduce fibrosis and the number of fibroblasts. It is well known that excess collagen deposition is a key factor in the formation of fibrosis. Masson staining was performed to detect the collagen content in fibrotic tissues, which revealed that the collagen content was significantly reduced in the IPFSC-EVs groups compared with the control group. In addition, the expression of collagens I and III was detected using immunohistochemistry. Our findings indicated that IPFSC-EVs could significantly suppress the expression of collagens I and III. Meanwhile, immunohistochemical analysis also revealed that IPFSC-EVs could reduce the expression of proinflammatory cytokines (IL-6 and TNF-*α*) and fibrosis marker (*α*-SMA). The above results suggested that IPFSC-EVs had the potential to inhibit the progression of knee arthrofibrosis.

In the in vitro study, the results of CCK8 and EdU cell proliferation assays demonstrated that IPFSC-EVs inhibited the viability and proliferative ability of fibroblasts in the inflammatory environment. Moreover, RT–PCR and western blot analyses confirmed that the expression of proliferation-related genes (*CCDN1* and *PCNA*) was decreased after treatment with IPFSC-EVs, which was in agreement with the aforementioned results. These results indicate that IPFSC-EVs could inhibit fibroblast proliferation in the inflammatory environment. Intriguingly, when *MT2A* was silenced in fibroblasts, the IPFSC-EV-induced inhibitory effect on cell proliferation was reversed.

This study was aimed at preliminarily exploring the inhibitory effect of IPFSC-EVs as a cell-free therapy on knee arthrofibrosis and screening and verifying the molecular targets of IPFSC-EVs to inhibit fibroblast proliferation in the inflammatory environment. However, this study is only a preliminary study and presents several limitations. First, IPFSC-EVs were administered via local injection, and although no adverse complications occurred in any animal, the safety and long-term effects of IPFSC-EVs need further experimental verification. Second, we did not explore which components in the EVs are involved in regulating cell proliferation. Third, because the situation in patients with severe knee arthrofibrosis is much more complex than in animal models, it remains unknown whether IPFSC-EVs can inhibit severe knee arthrofibrosis in clinical practice. Therefore, follow-up studies will be conducted in the future.

## 5. Conclusion

The findings of our study demonstrated that IPFSC-EVs can inhibit the progression of knee arthrofibrosis. This effect may be mediated by upregulating *MT2A* expression, which inhibits fibroblast proliferation in the inflammatory environment. Thus, IPFSC-EVs could be used as a novel therapeutic strategy to prevent knee arthrofibrosis following joint surgery.

## Figures and Tables

**Table 1:** Primer sequences for RT–PCR.

Genes	Primer sequence (5′–3′)
*MT2A*	S: CAACCTGTCCCGACTCTAGC
	AS: AGGTTTGTGGAAGTCGCGTT
*LVMT2A*	S: GATGTAAAGAACGCGACTTCC
	AS: GGAAGTCGCGTTCTTTACATC
*PCNA*	S: AGCCATATTGGAGATGCTGTTG
	AS: CTGAGTGTCACCGTTGAAGAGAG
*CCND1*	S: AGCTGTGCATCTACACCGAC
	AS: GAAATCGTGCGGGGTCATTG
*IL-6*	S: CAATGAGGAGACTTGCCTGGTG
	AS: TGGCATTTGTGGTTGGGTCA
*TNF-α*	S: ATGAGCACTGAAAGCATGATCC
	AS: AGGAGAAGAGGCTGAGGAACAAG
*GAPDH*	S: GGAAGCTTGTCATCAATGGAAATC
	AS: TGATGACCCTTTTGGCTCCC

S: sense; AS: antisence.

## Data Availability

The data used to support the findings of this study are available from the corresponding author upon request.
